# Mastering DNA chromatogram analysis in Sanger sequencing for reliable clinical analysis

**DOI:** 10.1186/s43141-023-00587-6

**Published:** 2023-11-13

**Authors:** Mohammed Baqur S. Al-Shuhaib, Hayder O. Hashim

**Affiliations:** 1 Department of Animal Production, College of Agriculture, Al-Qasim Green University, Al-Qasim 8, Babil, 51001 Iraq; 2 Department of Clinical Laboratory Sciences, College of Pharmacy, University of Babylon, Babil, 51001 Iraq

**Keywords:** Chromatogram files, Electropherograms, Sanger sequencing, Tips, Technical issues, Troubleshoot

## Abstract

**Background:**

Sanger dideoxy sequencing is vital in clinical analysis due to its accuracy, ability to analyze genetic markers like SNPs and STRs, capability to generate reliable DNA profiles, and its role in resolving complex clinical cases. The precision and robustness of Sanger sequencing contribute significantly to the scientific basis of clinical investigations.

**Main body of the abstract:**

Though the reading of chromatograms seems to be a routine step, many errors conducted in PCR may lead to consequent limitations in the readings of AGCT peaks. These errors are possibly associated with improper DNA amplification and its subsequent interpretation of DNA sequencing files, such as noisy peaks, artifacts, and confusion between double-peak technical errors, heterozygosity, and double infection potentials. Thus, it is not feasible to read nucleic acid sequences without giving serious attention to these technical problems. To ensure the accuracy of DNA sequencing outcomes, it is also imperative to detect and rectify technical challenges that may lead to misinterpretation of the DNA sequence, resulting in errors and incongruities in subsequent analyses.

**Short conclusion:**

This overview sheds light on prominent technical concerns that can emerge prior to and during the interpretation of DNA chromatograms in Sanger sequencing, along with offering strategies to address them effectively. The significance of identifying and tackling these technical limitations during the chromatogram analysis is underscored in this review. Recognizing these concerns can aid in enhancing the quality of downstream analyses for Sanger sequencing results, which holds notable improvement in accuracy, reliability, and ability to provide crucial genetic information in clinical analysis.

## Background

Sanger sequencing is one of the most important methods that are commonly used for DNA sequencing. Sanger sequencing, developed by Frederick Sanger in 1977 [1], represents a modified approach to DNA replication and stands as the initial technique regularly employed for DNA sequencing within laboratory settings. It relies on the incorporation of chain-terminating dideoxynucleotides and subsequent capillary electrophoresis to determine the nucleotide sequence of a DNA template. In Sanger sequencing, the DNA sequence is determined by synthesizing a complementary strand of DNA using a DNA polymerase enzyme, a template DNA strand, and a primer [2]. The differently labeled dideoxynucleotide allows a single reaction to be run in contrast to earlier methods relying on the same label for each chain termination, e.g., radiolabeling, and thus requiring 4 separate reactions. During the synthesis of the complementary strand, the DNA polymerase incorporates fluorescently labeled nucleotides at positions where they match the template DNA strand. Following synthesis, the newly formed DNA strands are size-separated through gel electrophoresis, with the sequence’s determination reliant on the color of the fluorescent label at each position. This process is facilitated by automated sequencing machines equipped with fluorescence detection capabilities [3]. Sanger sequencing holds extensive significance in medicine owing to its precision, dependability, and capacity to sequence minute quantities of DNA. This technique is effectively employed to analyze particular segments of mitochondrial DNA (mtDNA) and perform short tandem repeat (STR) analysis for the objective of human identification [4, 5]. Furthermore, Sanger sequencing serves to establish connections between biological evidence and individuals [6], identify genetic disorders [7], and investigate drug metabolism within the realm of forensic medicine [8].

Despite the existence of more advanced methods, Sanger sequencing remains the gold standard in various applications due to its reliability [9]. Being a first-generation DNA sequencing method, it deciphers the nucleotide sequence of DNA. While next-generation sequencing (NGS) methods offer high throughput advantages, Sanger sequencing maintains relevance for validating NGS outcomes due to its recognized accuracy, especially in routine low-volume synthetic DNA endeavors and similar applications. Even though Sanger sequencing enjoys extensive use and high accuracy, it remains susceptible to technical errors, particularly concerning the amplification and purification of PCR products preceding sequencing [10]. This review will delve into prevalent technical errors arising during PCR amplification and propose strategies to mitigate them, ensuring precise Sanger sequencing outcomes. A common source of technical errors in Sanger sequencing arises from non-specific amplification, leading to unintended DNA sequences that can compromise sequencing result accuracy and legibility. Adhering to sound laboratory practices, such as utilizing dedicated PCR workstations, employing sterile disposable pipette tips, and conducting PCR reactions in isolated rooms, helps counteract non-specific amplification. Moreover, the application of high-fidelity DNA polymerases with proofreading capabilities, capable of identifying and rectifying errors during DNA synthesis, aids in minimizing PCR-related errors [11]. Incomplete or incorrect PCR amplification of the target DNA template constitutes another potential origin of technical errors in Sanger sequencing. This situation can yield PCR products with inaccurate or partial sequences, introducing ambiguities or discrepancies in the ultimate sequencing outcomes. To avert this, optimizing PCR conditions—namely, annealing temperature, extension duration, and primer concentrations—is pivotal for ensuring efficient and precise amplification of the intended DNA template [12]. Employing multiple sets of overlapping primers is also advisable to guarantee comprehensive coverage of the entire target sequence and validate the accuracy of the eventual sequencing data. Furthermore, Sanger sequencing can encounter technical errors stemming from the utilization of subpar DNA templates or degraded DNA samples. These circumstances can manifest as low-quality sequencing traces and erroneous nucleotide determinations. To counteract this, employing well-preserved, high-quality DNA samples and subjecting them to quality control measures, such as quantification and gel electrophoresis, are vital steps for upholding DNA template integrity and purity [13].

The precision and dependability of Sanger sequencing findings hinge on the quality of the PCR products designated for sequencing. In order to preempt technical mishaps and attain precise sequencing results, adhering to sound laboratory practices, refining PCR conditions, and employing superior DNA templates are of paramount importance. Thus, ensuring the accuracy of Sanger dideoxy sequencing data encompasses several elements, underscoring the necessity for straightforward and reliable methods to monitor individual sequencing reactions.

Given its pivotal role in subsequent molecular biology experiments, the extensively utilized DNA sequencing methodologies demand vigilant attention to any nucleic acid variations. Owing to its feasibility and cost-effectiveness, Sanger DNA sequencing has found extensive application in pivotal domains other than clinical analysis, spanning biodiversity [14], animal production [15], microbial pathogenesis [16], medical genetics [17], and other recent applications, such as *in silico* computations [18]. Despite having developed multiple approaches to next-generation sequencing, Sanger biochemistry continues to serve as the foundation for sequencing production in numerous post-PCR genotyping protocols [19]. Sanger conventional sequencing has been fine-tuned to perform read lengths with high base accuracies as high as 99.999% [20]. Given its pivotal role in the majority of downstream molecular biology applications, any technical lapse in chromatogram interpretation can potentially undermine its validity [21].

To achieve reliable sequencing outcomes, sequencing chromatogram files should be downloaded and scrutinized. A chromatogram occasionally referred to as an electropherogram, visually illustrates the DNA samples produced by sequencing machinery, exemplified by systems like the Applied Biosystems ABI Sequence Detection System. The chromatogram corresponding to each DNA sample bears utmost significance and necessitates careful examination to authenticate any observed variations within the samples under investigation. Consequently, other files featuring different extensions, such as commonly encountered text data (with a .txt extension), should not be solely relied upon for interpreting sequencing data. Automated DNA sequencers generate four-color chromatograms that depict the sequencing run’s results, employing a computer program’s best approximation in deciphering the presented data [22]. Nonetheless, such computer programs do exhibit some errors, necessitating crucial manual cross-validation of computer-derived text data by cross-referencing it with chromatogram data. Predictable discrepancies within each chromatogram generally manifest at both the upstream and downstream segments of a sequencing run. Concurrently, other reading inaccuracies can manifest within the middle portion, compromising individual base calls or substantial data sections. Acknowledging the substantial impact of sequencing quality on subsequent analyses, multiple computer programs have been developed to detect, quantify, and comprehend errors stemming from Sanger sequencing pipelines. These computer-driven solutions play a pivotal role in enhancing the quality of sequencing runs. However, these automated algorithms might not comprehensively assess sequencing data quality. As a result, numerous computer-based tools remain susceptible to unidentified errors within chromatogram runs. Consequently, the assessment and analysis of sequencing run quality are imperative to ensure the credibility of nucleic acid variation reports.

Despite the numerous sequencing guidelines proposed over the past years to mitigate Sanger sequencing limitations [23], several challenges inherent in actual Sanger sequencing reads have not been adequately addressed. To the best of our knowledge, there is a lack of studies that comprehensively tackle the primary issues within sequencing interpretations in the existing literature [24]. In this review, we present straightforward and effective recommendations for identifying technical errors in the interpretation of various sequencing pipelines. These insights stem from our direct engagement with chromatograms. The adoption of these recommendations holds the potential to significantly mitigate a multitude of erroneous interpretations. The review accentuates prevalent problems linked to DNA sequencing, elucidating their potential origins, and corresponding remedies. While sequence data can encounter various issues, the delineated concerns are unequivocally the most frequently encountered across numerous sequencing endeavors. This endeavor is intended to aid researchers in scrutinizing the credibility of DNA sequencing chromatograms and to provide guidance on troubleshooting any challenges that might affect the interpretation of sequencing files.

## Main text

### Advantages and significance of the Sanger sequencing method in medicine

The importance of the Sanger sequencing method stems from a confluence of factors that collectively contribute to its practical success in downstream applications in medical sciences. Its attributes, including high accuracy, cost-effectiveness, compatibility with low-quality DNA, well-established methodology, and the capability to produce longer reads compared to alternative sequencing technologies, position it as the preferred choice across various genetic applications. The continued relevance of the Sanger sequencing method in DNA sequencing can be attributed to several key reasons: (1) accuracy: Sanger sequencing boasts remarkable precision, which renders it ideal for applications demanding highly reliable sequence data, such as clinical diagnostics and clinical analysis [25, 26]; (2) cost-effectiveness: despite the emergence of newer sequencing technologies like next-generation sequencing (NGS) that yield more data in less time, Sanger sequencing remains more cost-effective for specific applications [27]. For instance, it frequently finds utility in the targeted sequencing of particular genes or regions of interest, particularly when only a limited number of samples require sequencing; (3) compatibility with low-quality DNA: Sanger sequencing excels in generating high-quality sequence data even from subpar DNA samples, including degraded DNA from ancient or forensic sources; and (4) established methodology: having been employed for decades, the Sanger sequencing method possesses a well-recognized and widely understood methodology that resonates across the scientific community [28]. This facet facilitates the comparison and validation of sequence data produced by various laboratories. (5) Long reads: while Sanger sequencing cannot generate long reads like newer sequencing technologies, it can generate reads of up to 1000 base pairs, which can be useful for certain applications such as detecting structural variants and resolving challenging regions of the genome.

### Common software used in the reading of DNA chromatograms

Proficiency in interpreting DNA chromatograms stands as an indispensable competence within the realm of molecular biology and genetics research. The chromatograms from both the forward and reverse strands offer insightful revelations about the nucleotide sequence embedded within the scrutinized DNA fragment. This practice of deciphering chromatograms from both strands holds heightened significance, particularly in Sanger dideoxy sequencing, as it serves as a linchpin for the precision and dependability of sequencing data. Instances of misinterpretations, errors in base-calling, and artifacts might arise during sequencing endeavors, and their identification can prove especially formidable if solely reliant on the scrutiny of either the forward or reverse chromatogram. Numerous software tools are at one’s disposal to facilitate the scrutiny of Sanger sequencing chromatograms. For instance, the software Sequencher by Gene Codes Corporation enables concurrent visualization and analysis of both forward and reverse chromatograms. Correspondingly, Chromas, developed by Technelysium Pty Ltd., empowers users to peruse and manipulate chromatograms, discerning disparities and inaccuracies. A plethora of specialized programs designed to facilitate DNA chromatogram interpretation are accessible, including 4Peaks (exclusive to Mac), SnapGene Viewer, FinchTV, Sequence Scanner (compatible with both PC and MAC), Chromas (exclusive to PC), and SnackVar [27]. Nonetheless, computer programs do not unfailingly make accurate nucleotide calls. Instances may arise when the computer erroneously identifies a nucleotide, whereas a human observer could discern a different nucleotide within the same chromatogram reading. On occasion, the computer might designate an ‘N’ for a call where a human observer would confidently opt for a more specific base call. Such inaccuracies can manifest even in regions of the gel that are seemingly error-free. It is essential to swiftly survey the gel for minute peaks, N calls, and any peaks or nucleotides that are irregularly spaced. Regardless of the software employed for interpretation, the comprehension of DNA chromatograms demands meticulous attention to detail and an in-depth familiarity with the sequencing process. Once again, the presence of human intervention is strongly recommended to rectify all potential errors, with a particular focus on pivotal sites of nucleotide polymorphisms.

### Technical issues in reading DNA chromatograms

Numerous technical challenges can exert an impact on the precision and fidelity of the sequencing data extracted from DNA chromatograms. One prominent technical hurdle during the interpretation of DNA chromatograms from Sanger sequencing is the presence of extraneous noise within the data. This noise has the potential to trigger errors in base calling and impede the differentiation between authentic signals and background interference. Furthermore, the interpretation of DNA chromatograms can be impeded by sequence artifacts, like stutter peaks, that can emerge due to polymerase slippage during replication [29]. These artifacts can be intricate to distinguish from authentic sequence data and are capable of inducing inaccuracies in sequence analysis. Additionally, another technical challenge arises from double peaks present within DNA chromatograms. These dual peaks might stem from genuine heterozygosity, technical anomalies, or the occurrence of double infections. Identifying the nature of these double peaks can prove demanding, and their presence can lead to inaccuracies during sequence analysis. This discourse will delve into numerous instances of these challenges, with particular emphasis on those that have the potential to significantly compromise the accuracy and dependability of sequencing data.

### Optimum and noisy peaks

**Fig. 1:**
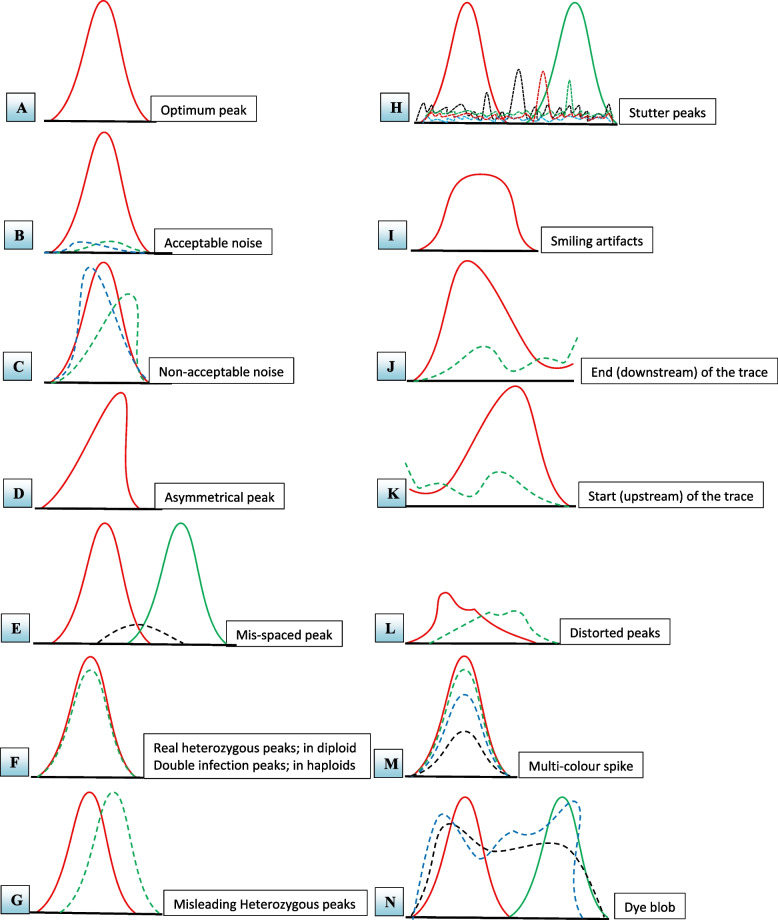
**A**–**N** The main issues encountered in the reading of DNA chromatograms of PCR products based on the Sanger sequencing method

Within an electropherogram, peaks symbolize the quantity of a specific molecule within the sample. Each peak’s dimensions—including its height, width, and form—yield vital insights into the sample, encompassing aspects like its purity, concentration, and size distribution. Optimal and noisy peaks in electropherograms serve as critical indicators of data quality and reliability derived from gel electrophoresis. Optimal peaks furnish precise and dependable insights into the size and quantity of analyzed molecules. In contrast, less optimal peaks may necessitate further refinement or troubleshooting to ameliorate data quality [30]. Optimal peaks exhibit distinct characteristics, including a well-defined shape, a sharply defined apex, and a robust signal-to-noise ratio (Fig. 1A). These peaks typically arise from a meticulously optimized electrophoresis protocol, where conditions are scrupulously controlled to ensure optimal separation and detection of target molecules. They are straightforward to interpret, with their heights directly correlating to sample molecule amounts. Furthermore, they are marked by clear resolution, symmetry, and a high signal-to-noise ratio. Well-resolved peaks indicate separation from adjacent peaks without overlap or smudging. Symmetrical peaks display a consistent form characterized by a smooth apex and uniform width on both sides. A high signal-to-noise ratio signifies that the peak’s height or area far exceeds the background noise level, signaling a robust and dependable signal. Optimal peaks are typically achieved by fine-tuning experimental conditions, a parameter that can vary depending on the specific application and the type of molecules under scrutiny. For instance, in DNA sequencing via capillary electrophoresis, optimal conditions encompass the utilization of high-quality DNA templates, meticulously refined PCR or sequencing primers, precise enzyme mixtures, reaction buffers, and suitable capillary electrophoresis operational settings [24]. Similarly, within real-time PCR, optimal conditions involve the selection of appropriate reference genes, optimally designed primers and probes, and standardized reaction parameters [13]. Conversely, suboptimal (or noisy) peaks exhibit a broad, rounded contour, a diminished signal-to-noise ratio, and an ill-defined peak apex. Often arising from less-than-ideal electrophoresis conditions, such as incorrect voltage, buffer composition, or temperature, these peaks prove more intricate to decipher. Their height may not correlate directly with the molecule quantity in the sample. The presence of minimal noise within a peak ensures uninterrupted calling (Fig. 1B). In certain instances, noisy peaks might even blend seamlessly with background noise or signals, rendering the extraction of meaningful data from the electropherogram challenging (Fig. 1C). Poorly resolved less optimum peaks appear indistinct and lack symmetry. This poor resolution might be traced back to multiple factors: insufficient separation or detection conditions, sample overload, or sample degradation or contamination. Asymmetrical peaks could be indicative of flaws in the electrophoresis setup or sample preparation, potentially leading to inaccurate size estimation or quantification of the molecules (Fig. 1D). A feeble signal-to-noise ratio could stem from substandard sample or reagent quality, inadequate detection sensitivity, or suboptimal operational conditions. However, with well-crafted PCR primers and a pristine DNA template, such noisy data could be minimized.

### Sequences artifacts

Sequence artifacts primarily stem from non-biological deviations within sequence data that result from the sequencing process rather than originating within the sample itself. These artifacts can emerge due to various factors, including inaccuracies in PCR amplification, the presence of inhibitors or contaminants, and DNA degradation during storage or handling [31]. Their existence can lead to inaccuracies during sequence analysis and can be challenging to differentiate from authentic sequence data. Moreover, sequence artifacts possess the potential to impact the precision of downstream analyses, such as variant calling, haplotype inference, and phylogenetic reconstruction. For instance, the presence of PCR errors might yield false-positive variants or inaccurate allele frequencies, consequently influencing analyses like association studies or clinical genetics [32]. Similarly, contamination can lead to the misidentification of microbial taxa or misconceptions about microbial diversity [33].

An effective approach to unearth artifacts or errors within a sequencing chromatogram is to meticulously scrutinize it, identifying misaligned peaks (Fig. 1E). Notably, incorrectly inserted nucleotides often manifest as oddly spaced in relation to neighboring bases. Misspaced peaks within DNA chromatograms can arise due to multiple factors, encompassing substandard DNA quality, PCR amplification errors, sequencing errors, and issues with electrophoresis or sequencing equipment [34]. Such mispaced peaks can engender erroneous base calling, consequently yielding flawed interpretations of genetic information. Therefore, comprehending the origins of mispaced peaks and their accurate interpretation is pivotal. PCR amplification errors stand as a common source of mispaced peaks, emerging from suboptimal reaction conditions, non-specific primer binding, or template DNA mutations. These errors might introduce additional peaks or obscure anticipated peaks within the chromatogram, rendering accurate genotype identification at specific loci a challenge. Inadequate sequencing chemistry or deficient base-calling algorithms can also give rise to such errors [35]. Further contributing to mispaced peaks are potential anomalies with electrophoresis or sequencing equipment. For instance, fluctuations in voltage or temperature within electrophoresis machines can yield artifacts or distortions in electropherograms, consequently leading to misaligned peaks. To ensure the accurate interpretation of mispaced peaks, it is pivotal to utilize high-quality DNA, optimize PCR conditions, and meticulously analyze chromatograms to pinpoint potential error sources [36]. Additionally, sequencing data should undergo a thorough assessment to ensure precise base calling and proper peak identification.

In light of the aforementioned, the recognition and mitigation of sequence artifacts can elevate the efficiency of data analysis and interpretation. Through their elimination, sequencing data quality can be augmented, curtailing the requirement for manual inspections and enhancing the accuracy of automated analysis pipelines. This can economize time and minimize the risk of human error.

### Zygosity detection or double infection potentials

Reading text files would likely yield inconclusive results, particularly when attempting to determine the zygosity (homozygosity/heterozygosity) potential of a specific peak of interest. Electropherograms typically exhibit a series of peaks, each corresponding to a specific nucleotide or fragment within the sample. In DNA sequencing, these peaks represent the sequence of nucleotides within the DNA template, while in fragment analysis, they signify fragment sizes. The configuration and height of these peaks offer critical insights into the analyzed sample. Significantly, the presence of homozygous and heterozygous peaks can reveal the genetic composition of the sample. Homozygous peaks denote singular nucleotides or fragment sizes, signifying that the individual carries two identical alleles at a given locus. In electropherograms, homozygous peaks appear as distinct, sharp peaks with a single peak height [37]. Conversely, heterozygous peaks indicate two distinct nucleotides or fragment sizes, revealing the presence of two different alleles at a given locus. These peaks appear broader and less defined, featuring two peak heights that correspond to the two alleles present within the sample [38]. Yet, direct reading of chromatograms can also unveil the zygosity status of sequenced DNA. This can be achieved by identifying double peak occurrences within the targeted locus. Double peaks characteristic of heterozygosity can be identified by observing a solitary peak position within a trace that manifests two distinct-colored peaks instead of just one (Fig. 1F). This is common when sequencing PCR products derived from diploid genomic DNA, where polymorphic positions concurrently exhibit both nucleotides. In such cases, one allele bears one nucleotide, while the other allele carries a different one. It is worth noting that the base caller may select the larger of the two peaks. Alternatively, the computer-based call may mark this as N or even favor the lower counterfeit peak. Herein, the human eye can provide a more precise interpretation. However, text sequences may fail to identify cases of two counterfeiting peaks, displaying only one nucleotide and disregarding the other. If all other sequences exhibit the same nucleotide reading in this specific locus, the zygosity status may go unnoticed unless chromatograms are manually examined. While it is feasible to scrutinize sequencing chromatograms for such heterozygous peaks in smaller projects, distinguishing between genuine and misleading heterozygous peaks remains essential. Genuine heterozygous peaks are readily distinguishable from misleading ones by examining the occupied position of the alternate peak. In authentic heterozygous peaks, both peaks are nearly aligned at the same baseline. In contrast, misleading heterozygous peaks exhibit distinct positions for each peak (Fig. 1G). This guide outlines the examination of normal DNA sequencing chromatograms, elucidating common issues and their interpretation. However, for extensive SNP-detection projects, specialized computer programs should be employed to detect such cases.

Detecting homozygous and heterozygous peaks within electropherograms holds significance for various genetic analyses, including genotyping and mutation detection. Accurate identification and interpretation of these peaks offer crucial insights into the genetic composition of individuals or populations. Notably, homozygous peaks in electropherograms signify a single allele, while heterozygous peaks signify two distinct alleles at a given locus. Thus, accurate detection and interpretation of these peaks are vital for a range of genetic analyses, including genotyping, mutation detection, and population genetics. The presence of homozygous and heterozygous peaks within electropherograms can assist in identifying an individual’s genotype at a specific locus. Mutations may alter DNA sequences, resulting in changes within electropherograms. The presence of homozygous and heterozygous peaks in electropherograms aids in identifying mutations linked to certain diseases or genetic disorders [39]. Moreover, the presence of these peaks facilitates the study of genetic diversity within and between populations. Consequently, precise peak interpretation is pivotal for accurate analysis and comprehension of genetic data [40].

In DNA chromatograms of haploid organisms, double peaks can sometimes indicate double infections, where multiple variants of the same genomic region coexist within the sample. Double infection can arise when different strains or isolates of a pathogen infect the same host or when a host is co-infected with multiple pathogens [41, 42]. Multiple peaks within chromatograms can complicate the analysis of sequencing data by hindering the accurate identification and quantification of genetic variants [43]. In some instances, double peaks may be misconstrued as heterozygosity or sequencing errors, leading to data misinterpretation [44]. Methods have been developed to differentiate between double peaks caused by double infection and other sources of variation. For instance, software tools like Novoalign are designed to specifically identify and segregate multiple strains or isolates within a sample [45]. This software can discern between double peaks arising from heterozygosity and double infections, aiding in their identification and separation. Alternatively, molecular methods such as polymerase chain reaction (PCR) or hybridization capture can selectively amplify or capture specific variants of a genomic region, enhancing the accuracy of quantification and identification of double infections [46]. While double infection may complicate sequencing data analysis, it offers valuable insights into the dynamics of pathogen populations and their interactions with host organisms. In particular, studying double infection can illuminate the mechanisms of coexistence and competition among different pathogen strains or isolates, informing disease control and prevention strategies.

#### Stutter peaks

Stutter peaks pose a common challenge in Sanger DNA sequencing analysis. These small peaks emerge at positions either immediately preceding or following the genuine signal peak, with their height constituting less than one third of the main peak’s height. Stutter peaks manifest as supplementary peaks spanning over two bases, appearing smaller than the main peak [47] (Fig. 1H). Such occurrences can introduce confusion during result interpretation, particularly when assessing low-quality samples. Various factors can contribute to stutter peaks, encompassing template secondary structure, and incomplete extension [48]. They often arise from DNA polymerase slippage, either during PCR amplification or within the sequencing reaction. Stutter peaks introduce complexity to sequencing data interpretation, potentially leading to incorrect base calling. This predicament is more pronounced in genomic areas harboring repetitive sequences or homopolymers, where distinguishing stutter peaks from authentic signals becomes more challenging [49]. Several strategies can mitigate the occurrence of stutter peaks in Sanger sequencing. Optimizing PCR conditions to curtail polymerase slippage is one approach, involving the use of higher fidelity polymerases or reducing the number of PCR cycles [50]. Additionally, the use of appropriate primer design can help reduce the occurrence of stutter peaks and improve the accuracy and reliability of the sequencing results. Utilizing software tools capable of identifying and subtracting stutter peaks from sequencing data is another avenue. Despite these efforts, stutter peaks can remain problematic in Sanger sequencing, particularly within intricate genomic regions [51]. Therefore, this issue should be recognized, and measures to mitigate its impact on data interpretation should be adopted.

### Reading limitations with larger size amplicons

The sequenced DNA template’s numbers represent the average signal strength of each nucleotide, ideally falling within the range of 200–400. Outside this range, researchers would anticipate obtaining 500–700 bases of reliably clean DNA sequences. For cases involving good DNA templates, DNA sequencers usually generate accurate data up to 900 or 950 nucleotides with minimal error rates. However, normal chromatograms eventually lose accuracy as they progress, marked by declining resolution, broader and shifting peaks, and increased difficulty in accurate base calling. The sequencer persists in attempting to “read” this data, but errors become progressively more frequent. As an example, upon further scrolling downstream in this chromatogram (towards higher-numbered nucleotides, such as around 900 nucleotides), the peaks exhibit broadening and diminished resolution (Fig. 1I). Nevertheless, the regular spacing between the top’s basecall letters often indicates data reliability. However, if the chromatogram is scrolled further and this spacing becomes irregular, only a few basecalls can be considered reliable, while others might be less reliable. The frequency of these errors increases as the reading progresses, and when encountered, it becomes necessary to disregard the remaining data using available editing tools. Sanger sequencing, widely used in DNA analysis, relies significantly on the length of the sequenced amplicons. As the amplicon’s length increases, the quality of the DNA chromatogram, a graphical representation of the sequencing data, can degrade. Various studies have explored the impact of amplicon size on DNA chromatogram resolution. One investigation noted a gradual reduction in resolution with growing amplicon size, with a noticeable drop in resolution for amplicons exceeding 800 base pairs (bp) [52]. The decline in resolution for larger amplicons in DNA chromatograms can be attributed to factors like incomplete primer extension, secondary structure formation, and heteroduplex formation. These factors contribute to peak intensity reduction and the emergence of broader peaks or “smiling” artifacts. To address Sanger sequencing’s limitations for larger amplicons, alternative sequencing technologies like NGS and single-molecule real-time (SMRT) sequencing have emerged [53, 54]. These technologies offer a superior ability to sequence longer DNA fragments with heightened resolution and accuracy compared to Sanger sequencing. Consequently, the decline in resolution for DNA chromatograms with larger amplicons is well-documented. While Sanger sequencing remains valuable, alternatives may be needed for larger amplicons.

### Limitations with smaller size amplicons

Reading DNA chromatograms of smaller-size amplicons presents several challenges due to various limitations. Amplicons with sizes under 100 base pairs often yield weaker signals that are challenging to differentiate from background noise. This complexity hampers the accurate reading of DNA sequences and the identification of potential mutations or variations. Particularly in smaller amplicons, interpreting chromatograms at the trace’s end is difficult. This area experiences a drop in signal intensity due to technology constraints or low DNA template abundance. In smaller amplicons, this drop might occur closer to the target sequence, making precise determination of the final nucleotide problematic. The low signal intensity at the trace’s end might resemble background noise, causing ambiguity in base calling (Fig. 1J). The same challenge applies to reading the trace’s start in the upstream readings of smaller amplicons (Fig. 1K). While both upstream and downstream traces are typically available for most amplicons, these extensions could significantly affect smaller amplicons, possibly spanning up to 50 nucleotides in length.

Another limitation of reading DNA chromatograms for smaller amplicons is the presence of overlapping peaks. This can create uncertainty in base calling and potentially misconstrue sequence data. Furthermore, smaller amplicons are more susceptible to PCR bias effects, which can lead to inconsistent amplification and sequence anomalies. PCR bias arises when specific DNA template regions are preferentially amplified, causing uneven read distribution across the amplicon and potentially skewing the resulting sequence data. To address these limitations, several strategies can be applied. Increasing the starting material or adopting more sensitive detection methods can enhance the signal-to-noise ratio. Optimizing PCR conditions, like using higher fidelity polymerases, reducing PCR cycles, or adjusting annealing temperatures, can help mitigate PCR bias and artifact formation. Some studies also recommend utilizing long-read single-molecule sequencing as a solution to these limitations [55]. Despite the constraints associated with reading DNA chromatograms of smaller amplicons, careful optimization and interpretation can yield valuable sequence data from these regions of interest. Recognizing these limitations and taking measures to mitigate their impact is essential for ensuring the accuracy and reliability of the data.

#### Distorted peaks

Chromatogram files can display distorted peaks due to various underlying factors. Several common culprits include contamination, column degradation, injection errors, solvent impurities, and instrument malfunctions. Contamination may arise during sample preparation or chromatography runs, leading to peak broadening, tailing, or splitting, ultimately distorting chromatogram peaks (Fig. 1L). Columns can degrade due to extended use, exposure to harsh conditions, or improper storage. Such degradation can induce peak broadening, splitting, or shifting, resulting in distorted peaks. Injection errors, like overloading the column, can also contribute to distorted peaks. Overloading can lead to peak broadening or splitting, while inadequate sample injection can cause peak tailing. Solvent impurities can disrupt the separation process and introduce peak distortion. Employing high-purity solvents and regularly replacing them is crucial. Instrument issues, including detector malfunctions, can similarly generate distorted peaks. Regular instrument maintenance and calibration can mitigate these problems. Hence, meticulous attention to factors such as sample preparation, column choice and upkeep, solvent purity, injection parameters, and instrument functionality is essential for minimizing the occurrence of distorted peaks in chromatogram files.

#### Multi-color spike

A multicolor spike observed in a DNA sequencing chromatogram refers to a concentrated peak that emerges as a multicolored spike within the sequence, typically obscuring only one or two nucleotides’ worth of data (Fig. 1M). This occurrence in a DNA sequencing chromatogram can indicate various potential issues connected to the sequencing reaction or the sequenced sample. The presence of a multicolor spike is usually attributed to an unusually high signal intensity for specific nucleotides or sets of nucleotides. Several potential causes for the appearance of a multicolor spike in a DNA sequencing chromatogram include dye blob, primer dimer, secondary structure, and contamination. A dye blob, for instance, results from the accumulation of excessive dye-labeled nucleotides during the sequencing reaction [56]. Such a blob can manifest as a multicolor spike in the chromatogram, frequently situated near the beginning of the read [34]. Dye blobs can introduce inaccuracies in base calling and may necessitate manual or software-based removal. Occasionally, the overall sequence might appear satisfactory except for a specific segment overshadowed by an exceptionally large peak. This peak structure is notably unusual and often arises due to erroneous dye incorporation during the sequencing procedure [57]. In certain instances, it might still be feasible to decipher the sequence beneath the “dye blob,” while in others, the blob renders the accurate determination of nucleotide bases implausible (Fig. 1N). Primer dimers are another factor that can materialize as a multicolor spike in the chromatogram, often at the outset of the read. Manual or software-driven data trimming can often address the presence of primer dimers. DNA template or primer secondary structure can hinder the sequencing reaction, resulting in stalling or misincorporation of nucleotides, thereby causing a multicolor spike in the chromatogram. Attentive primer design and optimization of sequencing conditions can mitigate the impact of secondary structure. Furthermore, contamination stemming from other DNA samples or residual DNA from prior samples can induce a multicolor spike in the chromatogram. The identification of contamination often involves assessing multiple sequencing reads from the same sample and may necessitate measures like re-preparation of the sample or decontamination procedures.

#### Reading indels

One of the paramount concerns within DNA chromatograms revolves around issues associated with the interpretation of indels, which can lead to inaccuracies in sequence reading [58, 59]. Indels, encompassing insertions and deletions, stand as a prevalent form of genetic variation capable of exerting substantial influence on gene structure and function [60]. Yet, discerning and accurately interpreting indels from DNA chromatograms poses challenges. A principal hurdle in reading indels from chromatograms lies in their capacity to induce shifts in the reading frame of the DNA sequence. Even minor insertions or deletions can thereby wholly modify the amino acid sequence of the resultant protein, rendering the prediction of functional consequences intricate. Moreover, discriminating indels from other sequencing errors, like base substitutions or homopolymer errors, presents difficulty [61], engendering the potential for false positives or negatives when attempting indel identification. Various studies have evaluated the precision of indel detection in DNA chromatograms. One such study contrasted the accuracy of four distinct software programs designed for indel detection in Sanger sequencing data, unveiling substantial variability in accuracy across different programs [62]. Another study assessed indel detection accuracy within next-generation sequencing data, revealing a pronounced influence of alignment algorithm choice and sequencing platform type [63]. Despite the intricacies tied to indel detection in DNA chromatograms, strategies have emerged to enhance precision. Utilizing multiple sequencing reads to corroborate indel presence constitutes one approach [64]. Additionally, employing specialized software tailored for indel detection stands as another strategy [65]. The precision of indel detection from chromatograms has seen advancements through various methods. Implementing dedicated software tools like PolyPhred or Mutation Surveyor, customarily designed for detecting indels and other sequence variations, is one such method [66]. Another approach involves employing a combination of sequencing technologies, such as both Sanger sequencing and next-generation sequencing, to elevate indel detection accuracy [67]. Despite the challenges, accurate indel detection and interpretation hold pivotal significance for comprehending the genetic underpinnings of diverse diseases and traits. Thus, ongoing research strives to refine the precision and reliability of indel detection methodologies.

## Conclusions

Sanger sequencing, a widely employed method for DNA sequencing, entails reading DNA sequences from chromatograms generated during sequencing. Nonetheless, interpreting DNA chromatograms can prove challenging in many clinical applications due to a range of technical intricacies that may result in misinterpretation of sequences. Among these complexities are noise, artifacts, and confusion between heterozygosity and double infection potentials, along with other errors. Thus, recognizing and resolving these technical challenges is pivotal to ensure accurate interpretation of DNA sequencing outcomes. This review delves into these technical aspects, serving as a resource for researchers and analysts striving to enhance the precision of DNA sequencing results and bolster downstream analyses. By addressing these technical issues, researchers can minimize errors and inconsistencies in subsequent analyses, thereby upholding the research findings’ fidelity. Given that troubleshooting unsuccessful sequencing reactions or problematic data hinges on in-depth chromatogram analysis, it is highly advisable to refer to the provided technical insights in case issues arise during data analysis. By preemptively considering these technical insights before interpreting chromatograms, researchers can proactively identify and rectify potential technical hurdles throughout the sequencing data analysis in clinical applications. This review serves as a valuable guide, aiding researchers in obtaining dependable and accurate DNA sequencing results, thus elevating the overall diagnostic quality in technical analysis. Hence, embracing these technical insights is crucial to safeguard the precision and credibility of DNA sequencing outcomes.

## Data Availability

All data generated or analyzed during this study are included in this published article.

## Abbreviations

mtDNA: Mitochondrial DNA
NGS: Next-generation sequencing
PCR: Polymerase chain reaction
SMRT: Single-molecule real-time
STR: Short tandem repeat
